# Molecular Mechanism of *Mok I* Gene Overexpression in Enhancing Monacolin K Production in *Monascus pilosus*

**DOI:** 10.3390/jof10100721

**Published:** 2024-10-16

**Authors:** Zhiwei Huang, Lishi Xiao, Wenlan Mo, Yaru Zhang, Yiyang Cai, Simei Huang, Zhiting Chen, Chuannan Long

**Affiliations:** 1 College of Food Science, Fujian Agriculture and Forestry University, Fuzhou 350002, China; xiao_lishi5258@163.com (L.X.); mowenlan2024@163.com (W.M.); azyr99999@163.com (Y.Z.); cai10404@126.com (Y.C.); huangsm0112@163.com (S.H.); a13950394763@163.com (Z.C.); 2 Fujian Provincial Key Laboratory of Quality Science and Processing Technology in Special Starch, Fujian Agriculture and Forestry University, Fuzhou 350002, China; 3 Jiangxi Key Laboratory of Bioprocess Engineering, Jiangxi Science and Technology Normal University, Nanchang 330013, China; longcn2004@126.com

**Keywords:** *Monascus pilosus*, monacolin K, *mok I* gene, overexpression, molecular mechanism

## Abstract

*Monascus* species are capable of producing various active metabolites, including monacolin K (MK) and pigments. Studies have shown that the overexpression of the *mok I* gene from the MK synthesis gene cluster in *Monascus* species can significantly increase MK production; however, the molecular mechanism has not yet been fully elucidated. Therefore, this study focused on the *mok I* gene of *Monascus pilosus* to construct overexpression strains of the *mok I* gene, resulting in high-yield MK production. Sixteen positive transformants were obtained, seven of which produced 9.63% to 41.39% more MK than the original strain, with no citrinin detected in any of the transformants. The qRT-PCR results revealed that the expression levels of *mok I* in the transformed strains TI-13, TI-24, and TI-25 increased by more than 50% compared to the original strain at various fermentation times, with the highest increase being 10.9-fold. Furthermore, multi-omics techniques were used to analyze the molecular mechanisms underlying enhanced MK production in transformed strains. The results indicated that *mok I* overexpression may enhance MK synthesis in *M. pilosus* by regulating the expression of key genes (such as *MAO*, *HPD*, *ACX*, and *PLC*) and the synthesis levels of key metabolites (such as delta-tocopherol and alpha-linolenic acid) in pathways linked to the biosynthesis of cofactors, the biosynthesis of unsaturated fatty acids, tyrosine metabolism, ubiquinone and other terpenoid-quinone biosynthesis, alpha-linolenic acid metabolism, and glycerophospholipid metabolism. These findings provide a theoretical basis for further study of the metabolic regulation of MK in *Monascus* species and for effectively enhancing their MK production.

## 1. Introduction

Red yeast rice (RYR) is a fermented product of *Monascus* species, capable of producing active components such as monacolin K (MK), pigments, and gamma-aminobutyric acid during the fermentation process [1,2,3]. Studies have shown that *Monascus*-fermented products, such as RYR, have significant lipid-lowering effects [4,5,6] and have gained recognition as highly regarded functional foods. RYR and its MK play a pivotal role in the improvement and treatment of atherosclerotic cardiovascular and cerebrovascular diseases [7,8,9]. MK, a secondary metabolite synthesized by polyketide synthase (PKS), belongs to the polyketone compound family [10], and is identical to lovastatin (LOV), synthesized by *Aspergillus terreus* [11]. The structure of MK is similar to that of 3-hydroxy-3-methyl-glutaryl-CoA (HMG CoA), which can competitively bind to HMG CoA reductase (HMGCR), thereby inhibiting cholesterol synthesis in the blood and reducing blood lipid levels [9,12]. MK can also induce the apoptosis of cancer cells. It enhances the sensitivity of cancer cells to chemotherapy drugs by affecting the expression of pathways such as MAPK, PI3K/AKT, and NF-κB in cancer cells, thus inducing apoptosis in acute myeloid leukemia, prostate cancer, breast cancer, lung cancer, gastric cancer, and liver cancer [12]. Shi et al. [13] found that MK can induce apoptosis in human glioma U251 cells by triggering ROS-mediated oxidative damage and regulating the MAPK and NF-κB pathways, without cytotoxicity to normal human cells, hUC-MSCs.

The MK synthesis pathway in *Monascus* species has been essentially elucidated today [14,15,16]. The MK synthesis gene cluster was isolated from the *Monascus pilosus* genome, comprising nine genes from *mok A* to *mok I*. The deduced amino acid sequences share over 54% similarity with the LOV synthesis gene cluster in *A. terreus*. For instance, the products of *lov B* and *lov F* in *A. terreus* show homology of 76% and 73%, respectively, with the products of *mok A* and *mok B* of *M. pilosus*, featuring several similar specific functional domains (reductase, acyl transferase, ketone synthase, etc.) [17]. In the MK synthesis gene cluster, *mok A* encodes a PKS that primarily facilitates the synthesis of the main chain structure of MK, functionally similar to the lovastatin nonaketide synthase (LNKS) in *A. terreus* [17]. The product of *mok H* acts as a transcription factor, and overexpression of *mok H* significantly enhances MK synthesis capability [11]. *mok B* encodes a PKS similar to lovastatin diketide synthase (LDKS), accelerating the synthesis of the diketide portion of the side chain of MK [18]. Zhang et al. [19] applied the protoplast electroporation transformation of *M. pilosus* to construct overexpression strains of *mok C*, *mok D*, *mok E*, and *mok I* and found that MK production increased by 234.3%, 220.8%, 89.5%, and 10%, respectively, compared to the original strains. Huang et al. [20] knocked out the *lov F* gene to disrupt lovastatin biosynthesis, constructed a high-yield monacolin J (MJ) strain, and then knocked out the *lov E* gene in the strain for overexpression under the control of the *PgpdAt* promoter, increasing the MJ content by 52.5%.

Efflux pumps, proteins located in the cell membrane, play a crucial role in transporting substances across the membrane by consuming energy. The genes encoding efflux pumps have significant effects on the metabolic regulation of microorganisms [21]. These pumps are responsible for actively expelling toxic compounds from within the cell to the external environment, forming a protective mechanism [22]. The increased activity of efflux pumps has been observed to alter the metabolic rate, promoting material transformation, particularly at very high intracellular solvent concentrations, where expulsion helps prevent internal cell damage [23]. Ley et al. [24] demonstrated that the active export of statins from yeast reduced toxicity. This was achieved by integrating the efflux pump-encoding gene *mlc E* from a mevastatin-producing *Penicillium* into the brewing yeast genome. All resulting strains, when compared with wild-type strains, exhibited increased resistance to natural statin drugs.

In summary, the genomic sequence of the MK synthesis gene cluster in *M. pilosus* has been reported [17]. Various studies have explored the functions of *mok A* [17], *mok B* [18], and *mok H* [11]. However, research on the function of *mok I* is relatively limited, and the molecular mechanism by which *mok I* overexpression increases MK production in *Monascus* species has not yet been elucidated [19,25]. In recent years, high-throughput detection and analysis methods, such as transcriptomics and metabolomics, have become important tools for studying fungal growth, development, and secondary metabolism regulation mechanisms. Significant advances have been made in understanding secondary metabolism in *Monascus* species [26,27,28]. This study specifically targeted the efflux pump gene *mok I* within the MK synthesis gene cluster of *M. pilosus*, aiming to construct engineered strains with *mok I* overexpression to enhance MK production. Additionally, the study aimed to comprehensively analyze the molecular mechanisms by which *mok I* overexpression increases MK production in *M. pilosus* using multi-omics methods.

## 2. Materials and Methods

### 2.1. Strains and Media

The *M. pilosus* strain CICC 5045 was procured from the China Center of Industrial Culture Collection (Beijing, China). *Agrobacterium tumefaciens* strain AGL-1 was generously provided by Dr. Xue Yong from Xiamen University (Xiamen, China). Competent cells of *Escherichia coli* strain Top 10 were obtained from Beijing Ding Guo Chang Sheng Biotechnology Co., Ltd. (Beijing, China).

Luria Bertani (LB) Medium [29] (used for cultivating *E. coli*) consisted of 5 g/L yeast extract, 10 g/L peptone, 10 g/L NaCl, pH 7.0. Fifteen g/L agar powder was added when preparing the solid medium.

Yeast Extract (YEB) Medium [29] (used for cultivating *A. tumefaciens*) consisted of 1.0 g/L yeast extract, 5.0 g/L peptone, 5.0 g/L beef extract, 4.0 g/L MgSO_4_·7H_2_O, pH 7.0. Fifteen g/L agar powder was added when preparing the solid medium.

Potato Dextrose Agar (PDA) medium [29] (used for cultivating *M. pilosus* strains and their geneticin susceptibility assay) is prepared as follows: After peeling the potatoes, weigh 200 g, cut into small pieces, boil for 30 min, filter through eight layers of gauze, add 20 g agar powder and 20 g glucose, dissolve, and add water to make up to 1 L.

The Auto-induction Medium (AIM) [30] (used for co-cultivation of *A. tumefaciens* and *M. pilosus*) was composed of the following components: 0.6 g MgSO_4_·7H_2_O, 0.3 g NaCl, 1 mL FeSO_4_ (1 mg/mL), 40 mL 2-morpholine ethanesulfonic acid (MES, pH 5.5), 1 mL CaCl_2_ (1%), 1 mL (NH_4_)_2_SO_4_ (0.33 g/L), 10 mL glycerol (50%), and 0.8 mL potassium phosphate buffer (pH 4.8, 1.25 mol/L); 5 mL microelement stock solution (0.1 g/L) which consisted of Na_2_MoO_4_·2H_2_O, H_3_BO_3_, MnSO_4_·H_2_O, ZnSO_4_·7H_2_O, CuSO_4_·5H_2_O; glucose 2 g/L (used for liquid media) or 1 g/L (used for solid media), pH 5.4. 15 g/L agar powder was added when preparing the solid medium.

The AIM induction medium [30] (used for co-cultivation of *A*. *tumefaciens* and *M. pilosus*) was prepared as follows: 78.5 mg of acetosyringone (AS, 0.2 mmol/L) was added to 2 mL of dimethyl sulfoxide solution to prepare a 0.2 mol/L stock solution. Then, 0.2 mol/L AS solution was added to the AIM medium until the final concentration reached 0.2 mmol/L.

Screening medium [30] (used for screening cultivation of *M. pilosus* transformants) consisted of PDA medium with 80 mg/L Geneticin G418, 200 µmol/L cefotaxime, and 0.2% Triton X-100.

The seed liquid culture medium [31] (used for liquid culture of *M. pilosus* spores) consisted of 30 g/L glucose, 10 g/L peptone, 70 g/L glycerol, 2 g/L KH_2_PO_4_, 15 g/L fermentation-specific soybean meal powder, 2 g/L NaNO_3_, and 1 g/L MgSO_4_·7H_2_O.

The liquid fermentation medium [31] (used for submerged fermentation of *M. pilosus* strains) consisted of 90 g/L glycerol, 10 g/L peptone, 2.5 g/L KH_2_PO_4_, 1 g/L MgSO_4_·7H_2_O, 2 g/L ZnSO_4_·7H_2_O, and 1 g/L NaNO_3_.

For the solid fermentation medium (used for solid-state fermentation of *M. pilosus* strains), 30 g of rice was placed in a plastic fermentation bottle (350 mL), and 30 mL of a mixture of water and glycerol (4:1, *v*/*v*) was added.

### 2.2. Construction of a Eukaryotic Expression Vector for Mok I Gene

The *mok I* gene was synthesized based on the nucleotide sequence of *mok I* in the MK synthesis gene cluster (GenBank: DQ176595.1) of *M. pilosus* strain BCRC38072 and cloned into the pMD^®^18-T Simple Vector (Takara Biomedical Technology Co., Ltd., Dalian, China) by Boshang Biotechnology Co. (Shanghai, China). The constructed plasmid was designated as pMD-mkI. Subsequently, the plasmid pMD-mkI was digested using *Hind* III and *Sac* I, and the *mok I* gene fragment was recovered and ligated to the expression vector pNeo-0380 [32].

Using the heat shock method [29], the ligation products were transformed into the competent cells of *E. coli* Top 10 and plated on LB medium containing 50 µg/mL Kanamycin (Kan). Single colonies were selected after inverted cultivation at 37 °C for 16 to 20 h and inoculated into LB liquid medium containing 50 µg/mL Kan. After shaking at 220 r/min at 37 °C for 16 h, the plasmid DNA was extracted from the culture using the E.Z.N.A^®^ Plasmid Mini Kit I (Omega, Norcross, GA, USA). The obtained plasmid DNA was stored in a −20°C freezer for future use.

Primers mkI2-8 (5′-CAATTTGGCTCAGACCCATCAT-3′) and PgpdA-F1 (5′-CTGCACTCGACCTGCTGAGGTC-3′) were designed based on the nucleotide sequence of *mok I* and the promoter *PgpdA* using the online program Primer3web version 4.1.0 (https://primer3.ut.ee/), and synthesized by Fuzhou Boshang Biotechnology Co., Ltd., China. PCR identification of the extracted plasmid DNA was performed using 2 × Fine Taq PCR SuperMix (+dye) (TransGen Biotech, Beijing, China). Subsequently, the extracted plasmid DNA was verified through *Hind* III/*Sac* I and *Bam*H I/*Sac* I double digestion to obtain the recombinant expression vector pNeo-mkI (Figure S1). The volume of the PCR identification reaction system was 10 µL, which contained 5 µL 2×Fine Taq^TM^ PCR SuperMix (+dye), 0.2 µL mkI2-8 (20 µM), 0.2 µL PgpdA-F1 (20 µM), 0.8 µL plasmid DNA of the transformed *E. coli*, and 3.8 µL ddH_2_O. After mixing the reaction system, PCR reaction was carried out under the following conditions: 94 °C for 5 min; followed by 35 cycles of 94 °C for 30 sec, 58 °C for 30 sec, 72 °C for 50 sec; and 72 °C for 10 min. The volume of the digestion verification reaction system was 30 µL, consisting of 5 µL Quick Buffer, 1 µL *Hind* III or *Bam*H I, 1 µL *Sac* I, 0.8 µL plasmid DNA from the transformed *E. coli*, and 24.2 µL ddH_2_O. The reaction condition for *Hind* III/*Sac* I double digestion was 37 °C for 15 min, and that for *Bam*H I/*Sac* I double digestion was 30 °C for 5 min first, followed by 37 °C for 15 min.

### 2.3. Geneticin Susceptibility Assay for M. pilosus

*M. pilosus* CICC 5045 was inoculated onto PDA plates containing *Geneticin* G418 at concentrations of 40, 50, 60, 70, 80, and 90 µg/mL, respectively, and cultured at 28 °C for 5–7 days under dark conditions in a constant-temperature incubator.

### 2.4. Agrobacterium-Mediated Transformation of M. pilosus

The constructed recombinant expression vector pNeo-mkI was transformed into *A. tumefaciens* AGL-1 using the freeze–thaw method [29]. Following the method described in Section 2.2, the obtained *A. tumefaciens* transformants were identified by PCR and digestion, resulting in the recombinant *A. tumefaciens* pNeo-mkI/AGL-1 with the recombinant expression vector. The obtained recombinant *A. tumefaciens* was stored in a −80 °C freezer for the following use.

Co-culture and screening of *M. pilosus* and *A. tumefaciens* was performed as follows: The recombinant *A. tumefaciens* pNeo-mkI/AGL-1 was inoculated into 20 mL of liquid YEB medium (containing 20 µg/mL Kan and 20 µg/mL rifampicin) and cultured at 28 °C with shaking at 220 r/min for 48 h. The *A. tumefaciens* solution was then diluted with AIM induction medium and 200 µmol/L AS to an OD_600_ of 0.15–0.16 and cultured at 28 °C with shaking at 220 r/min for 6 h. *M. pilosus* spore conidia were filtered and diluted to a concentration of 1 × 10^6^/mL. The *A. tumefaciens* and spore suspension were mixed in a 1:1 ratio, applied onto AIM induction medium plates, and incubated in the dark at 28 °C for 48 h. Screening medium (PDA medium containing 80 µg/mL G418, 200 µmol/L cefotaxime, and 0.2% Triton X-100) was added onto the AIM induction medium, and screening was conducted for 3–7 days. Resistant transformants were selected, and screening culture was continued for three generations.

### 2.5. PCR Identification of M. pilosus Transformants

The resistant transformants of *M. pilosus* were inoculated into liquid malt broth medium (Qingdao Hope Bio-Technology Co., Ltd., Qingdao, China) and cultured at 28 °C with shaking at 220 r/min for 4 days, using the original strain CICC 5045 as a control. The cultured mycelium was filtered through filter paper, placed a mortar, and ground into powder with liquid nitrogen. Genomic DNA was extracted from the mycelium of resistant transformants using a novel rapid genomic DNA extraction kit (Beijing Ding Guo Chang Sheng Biotechnology Company, Ltd., Beijing, China) according to its instructions. The obtained genomic DNA was stored in a −20 °C freezer for later use.

PCR identification of *M. pilosus* transformants was performed using 2 × Fine Taq PCR SuperMix (+dye) (TransGen Biotech, Beijing, China), with the genomic DNA of transformants as templates and the primers mkI 2-8 and PgpdA-F1. The reaction system and conditions for PCR identification were referred to in Section 2.2.

### 2.6. HPLC Detection of MK Yield of the Transformed Strains

#### 2.6.1. Solid Fermentation of the Transformed Strains

The positive transformation strains TI-11~TI-26 identified by PCR and the original strain CICC 5045 were respectively inoculated on PDA plates for activation culture for 7 days. They were then inoculated into seed liquid culture medium and cultured at 28 °C with shaking at 160 r/min for 48 h. The conidia were filtered using sterilized wiping paper and diluted with sterile water to a concentration of 1 × 10^6^/mL.

The spore suspensions of different strains were separately inoculated into solid-state fermentation (SSF) medium at an inoculation rate of 10%. This procedure was repeated four times for each strain, followed by culturing at 28 °C for 15 days. After fermentation, RYR samples were placed in a 50 °C drying oven, dried to a constant weight, and ground into powder for later use.

#### 2.6.2. HPLC Detection Method for MK Content

High-performance liquid chromatography (HPLC) was employed for detection using the Waters e2695 HPLC System (Waters, Milford, MA, USA). The HPLC detection conditions were as follows: Waters SunFire C18 (5 μm, 4.6 mm × 150 mm, No. 186002559) chromatographic column, acetonitrile—0.1% phosphoric acid solution (55:45, *v*/*v*) as the mobile phase, 1 mL/min flow rate, 238 nm UV detector wavelength, 30 °C column temperature, and 20 μL sample volume.

For the preparation of the acidic MK standard solution, 1.5 mg of MK standard sample was accurately weighed and poured into a 10 mL brown volumetric flask. Then, 2 mL of 0.2 mol/L NaOH solution was added for treatment, and the solution was diluted to 10 mL with 75% ethanol. The mixture was placed in a 50 °C water bath for 35 min, followed by ultrasonic treatment for 35 min to prepare a standard solution with a concentration of 150 μg/mL.

To prepare the 150 μg/mL lactone MK standard solution, 1.5 mg of MK standard sample was accurately weighed and poured into a 10 mL brown volumetric flask, dissolved in 75% ethanol, and diluted.

Test samples of transformation strain fermentation products were prepared as follows: First, 1.0 g of RYR sample powder was weighed and poured into a 10 mL brown volumetric flask. Then, a 75% ethanol solution was added to reach a volume of 10 mL and the mixture was treated with ultrasound at 30 °C for 40 min. It was shaken well once during and after ultrasonic treatment and allowed to stand for 15 min. The supernatant was then collected and filtered through a 0.45 μm filter membrane for sample injection.

After HPLC detection, the content of acid MK and lactone MK (mg/kg) in the tested sample was separately calculated. The calculation equation for MK content was as follows: standard sample concentration × (sample chromatographic peak area/standard sample peak area) × fixed volume/mass of the tested sample.

### 2.7. Mass Spectrometry Identification of MK in Transformed Strains

Detection was performed using HPLC-quadrupole time-of-flight tandem mass spectrometry (Q-TOF). The mobile phase consisted of 0.1% formic acid in water—acetonitrile (45:55, *v*/*v*). The flow rate, chromatographic column, detection wavelength, and column temperature were as described in Section 2.6.2. The sample volume was 10 μL. The nebulizing gas pressure was set at 40 psi, the drying gas flow rate was 10 L/min, and the drying gas temperature was maintained at 350 °C. ESI (+) mode was adopted, and the scanning range was 200–800 *m*/*z*.

### 2.8. HPLC Detection of Citrinin Content in Transformed Strains

First, 1.0 g of RYR sample powder was weighed and placed in a volumetric flask. Then, 80% methanol was added to reach a final volume of 5 mL. The mixture was subjected to ultrasonic treatment for 30 min, shaken well, and soaked at 60 °C with shaking at 200 r/min for 1 h. After centrifugation at 8000 g for 15 min, the supernatant was collected and filtered through a 0.22 μm filter membrane for sample injection detection.

HPLC detection was performed under the following conditions: acetonitrile—0.1% phosphoric acid solution (50:50, *v*/*v*) mobile phase, pH 2.6 adjusted with phosphoric acid or acetic acid, and detection wavelength of λ ex = 331 nm, λ em = 500 nm; chromatographic column, flow rate, column temperature, and sample volume were as described in Section 2.6.2.

### 2.9. Detection and Analysis of Expression Levels of Mok I Gene in Transformed Strains

Following the method described in Section 2.6.1, the transformed strains TI-13, TI-24, and TI-25, which exhibited higher MK production, were selected for SSF for 4, 8, and 12 days, respectively, using the original strain CICC 5045 as a control. Three replicates were set up for each treatment. After completing the SSF, *M. pilosus* spores were isolated from the fermentation product following the method of Wang et al. [33]. Then, total RNA was extracted from the spores following the method of Huang et al. [6]. and detected by agarose gel electrophoresis. The reverse transcription (RT) reaction was conducted to synthesize the first strand of cDNA using total RNA as templates and the *TransScript^®^* One-Step gDNA Removal and cDNA Synthesis SuperMix (TransGen Biotech Co., Ltd., Beijing, China).

The relative expression levels of the *mok I* gene in the tested samples were detected and analyzed using a real-time fluorescent quantitative PCR (qRT-PCR) instrument (ABI QuantStudio 3, Thermo Fisher, Singapore City, Singapore) following the method of Huang et al. [34]. The sequences of primers (Table S1) required for qRT-PCR were referenced from the literature [35].

### 2.10. Multi-Omics Analysis of the Molecular Mechanism of High MK Production in Transformed Strains

#### 2.10.1. Transcriptomic Analysis of the Molecular Mechanism of High MK Production in Transformed Strains

The total RNA from the transformed strain TI-25 and the original strain CICC 5045, obtained in Section 2.9, was used for cDNA library construction and RNA sequencing. The sequencing data were then subjected to transcriptomic analysis following the method described by Huang et al. [6]. The screening threshold for differentially expressed genes between groups was set at FDR < 0.05 & FC ≥ 1.5 or FC ≤ 1/1.5.

#### 2.10.2. Metabolomic Analysis of the Molecular Mechanism of High MK Production in Transformed Strains

First, 50 mg of RYR samples from the transformed strain TI-25 and the original strain CICC 5045 after SSF in Section 2.9 were weighed. Six replicates were set up for each treatment and placed in a 2 mL centrifuge tube. A 6 mm diameter grinding bead and 400 μL of extraction solution (methanol:water = 4:1, *v*/*v*), containing 0.02 mg/mL of four internal standards, including L-2-chloroalanine, were added. The samples were ground with a frozen tissue grinder for 6 min (−10 °C, 50 Hz). After grinding, the samples were pre-processed, and LC-MS detection and metabolomic analysis of the data were performed following the method of Huang et al. [6]. The screening thresholds for inter-group differential metabolites were set at FDR < 0.05 & VIP > 1 & |log2FC| ≥ 1.

#### 2.10.3. Multi-Omics Joint Analysis of the Molecular Mechanism of High MK Production in Transformed Strains

The differential genes and metabolites between the transformed strain TI-25 and the original strain CICC 5045 were jointly analyzed using the Meiji Cloud Platform (www.majorbio.com) following the method of Han et al. [36]. The key metabolic pathways that enhance MK production in *M. pilosus* through the overexpression of the *mok I* gene were identified by cross-validating the transcriptomics and metabolomics data, leading to an analysis of the molecular mechanism underlying the high MK production.

## 3. Results

### 3.1. Obtaining Transgenic Strains Overexpressing Mok I

#### 3.1.1. Construction of Eukaryotic Expression Vectors for *Mok I*

The band sizes obtained from PCR identification and enzyme digestion verification of the recombinant plasmid pNeo-mkI were consistent with the predicted values (Figure S2), indicating the successful construction of the eukaryotic expression vector pNeo-mkI for *mok I*.

#### 3.1.2. Results of Agrobacterium-Mediated Transformation of *M. pilosus*

Following the transformation of the recombinant plasmid pNeo-mkI into *A. tumefaciens* AGL-1, the PCR identification and enzyme digestion verification results of the *A. tumefaciens* transformations are illustrated in Figure S2. This confirms that the recombinant *A. tumefaciens* pNeo-mkI/AGL-1 carrying the recombinant plasmid has been successfully obtained.

The G418 sensitivity identification results of *M. pilosus* demonstrated that strain CICC 5045 exhibited robust growth on PDA plates containing 40 or 50 µg/mL G418, while minimal growth was observed on PDA plates containing 60–90 µg/mL G418. Consequently, a G418 concentration of 80 µg/mL was chosen for screening the transformants of *M. pilosus*.

#### 3.1.3. PCR Identification Results of *M. pilosus* Transformants

Following the co-cultivation of *M. pilosus* spores and recombinant *A. tumefaciens*, a total of 57 resistant transformants were acquired through the screening cultivation process. The PCR identification results of *M. pilosus* transformants (refer to Figure S3) confirmed the successful generation of 16 positive transformants designated as TI-11 to TI-26.

### 3.2. Results of MK Content Detection in Transformed Strains

**Figure 1:**
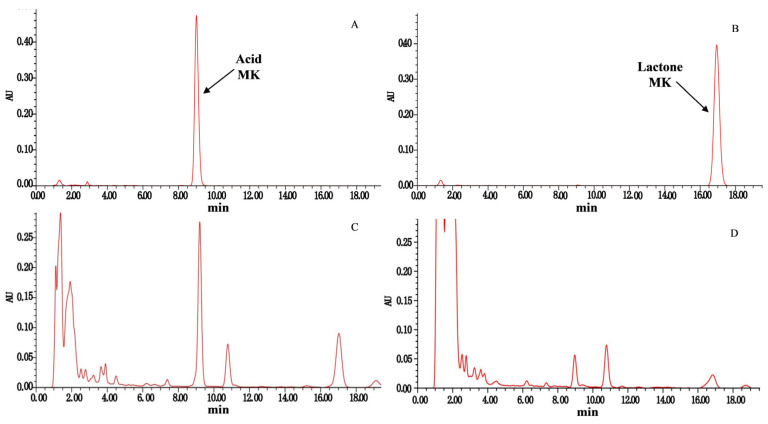
HPLC chromatograms of MK from the transformed strains and the original strain of *M. pilosus*. (**A**) Acidic form of MK standard. (**B**) Lactone form of MK standard. (**C**) Samples of transformed strains of *M. pilosus*. (**D**) Sample of the original strain of *M. pilosus*.

**Figure 2:**
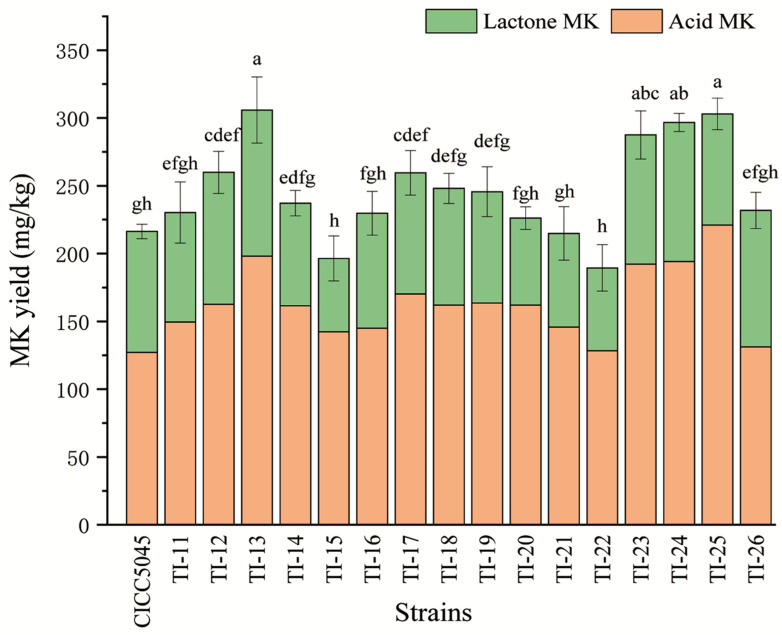
MK yield of the transformed strains and the original strain of *M. pilosus*. Different letters in different strains indicate significant differences (*p* < 0.05).

The HPLC chromatograms presented in Figure 1 depicted the samples collected from transformed strains and the original strain of *M. pilosus*. Figure 2 illustrates the MK yield in RYR fermented by different strains. The total MK yield, comprising both acid MK and lactone MK, was notably higher in seven transformed strains (TI-12, TI-13, TI-17, TI-18, TI-23, TI-24, and TI-25) compared to the original strain. Notably, strains TI-13, TI-24, and TI-25 exhibited significant increases in MK yield, registering at 41.39%, 37.15%, and 40.08%, respectively. Conversely, the total MK yields of the remaining nine transformed strains (TI-11, TI-14, TI-15, TI-16, TI-19, TI-20, TI-21, TI-22, and TI-26) showed no significant difference compared to the original strain.

### 3.3. Mass Spectrometry Identification Results of MK in Transformed Strains

**Figure 3:**
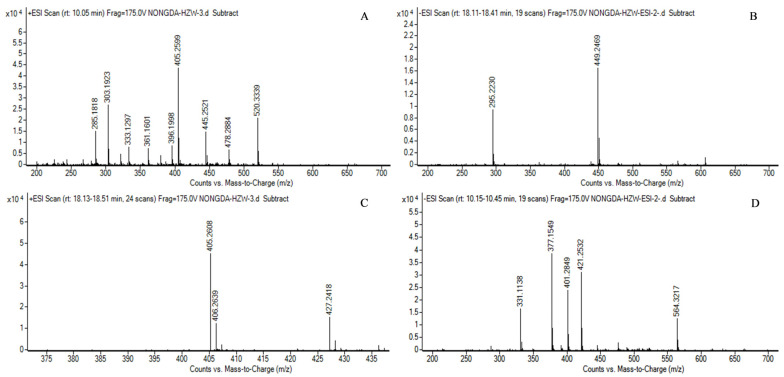
Identification of MK in the samples of transformed strain by HPLC/MS. (**A**) Mass spectrogram of lactone MK standard under positive ion mode (M + H)^+^ and (M + Na)^+^. (**B**) Mass spectrogram of lactone MK standard under negative ion mode (M + COOH)^−^. (**C**) Mass spectrogram of acid MK in the sample of the transformed strain under positive ion mode (M + Na)^+^; (**D**) Mass spectrogram of lactone MK in the sample of the transformed strain under negative ion mode (M − H)^−^.

The HPLC-Q-TOF analysis results depicted in Figure 3 revealed the presence of both acid MK and lactone MK in the fermentation products of the transformed strain TI-25. This identification further validated that the overexpression of *mok I* enhanced the MK synthesis capability of *M. pilosus*, as indicated by the aforementioned HPLC detection results.

### 3.4. HPLC Test Results of Citrinin Content in Transformed Strains

**Figure 4:**
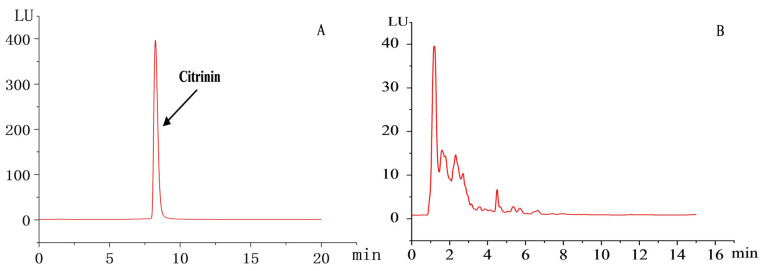

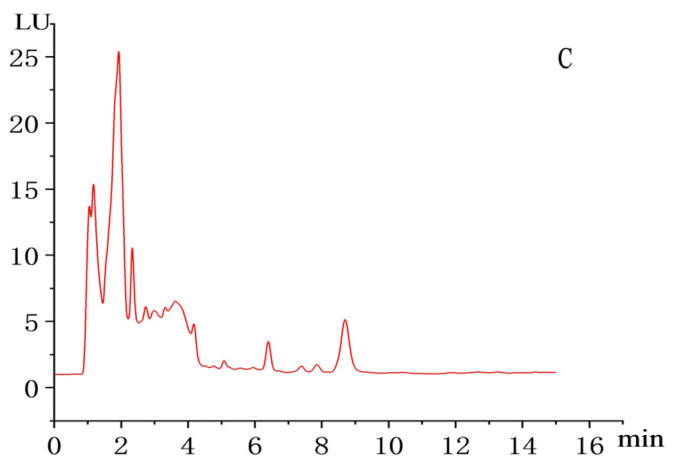
HPLC chromatograms of citrinin from the transformed strains of *M. pilosus* and commercially available RYR. (**A**) Citrinin standard. (**B**) Transformed strain of *M. pilosus* samples. (**C**) Commercially available RYR samples.

The chromatogram presented in Figure 4 illustrated the results of citrinin content detection. The test sample of commercially available RYR exhibited the presence of citrinin (Figure 4C), whereas the test sample of the transformed strain TI-25 did not show detectable levels of citrinin. This suggested that citrinin production in RYR fermented by the transformed strain TI-25 either fell below the detection limit or did not occur.

### 3.5. Detection Results of Expression Levels of Mok I Gene in Transformed Strains

**Figure 5:**
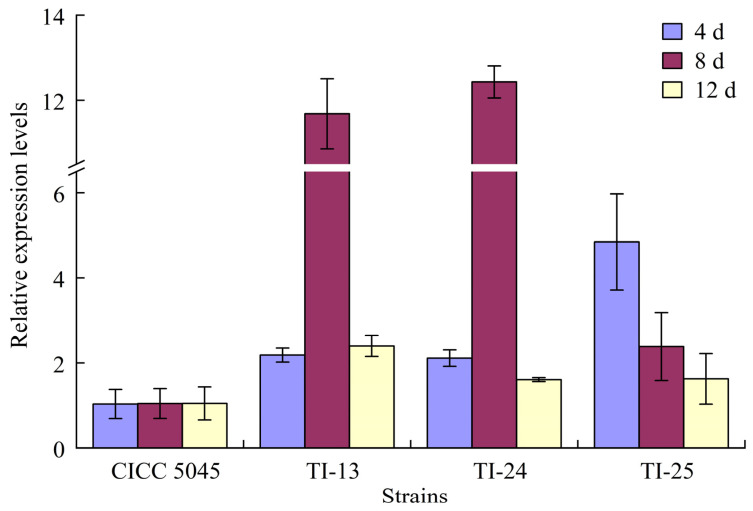
Expression levels of *mok I* gene in the transformed strains and the original strain.

The qRT-PCR detection results, as depicted in Figure 5, showed that the expression levels of the *mok I* gene in the transformed strains TI-13, TI-24, and TI-25 increased by 1.1-fold, 1.0-fold, and 3.7-fold, respectively, compared to the original strain after 4 days of fermentation. After 8 days of fermentation, these levels increased by 10.2-fold, 10.9-fold, and 1.3-fold, respectively. After 12 days of fermentation, the increases were 1.3-fold, 53.2%, and 55.1%, respectively. These results confirmed the successful overexpression of *mok I*, particularly with a significant increase in expression levels after 8 days of fermentation, which promoted MK synthesis in *M. pilosus* and led to a substantial increase in MK production.

### 3.6. Transcriptomic Analysis Results of the Molecular Mechanism of High MK Production in Transformed Strains

#### 3.6.1. Differential Gene Expression Analysis of Transformed Strains

The RNA quality testing results (Table S2) indicated that all RNA samples met the requirements for library construction and RNA sequencing. The RNA sequencing results revealed a total of 7059 expressed genes in the TI-25 group of the transformed strain and the CK group of the original strain at 4, 8, and 12 days of fermentation.

**Figure 6:**
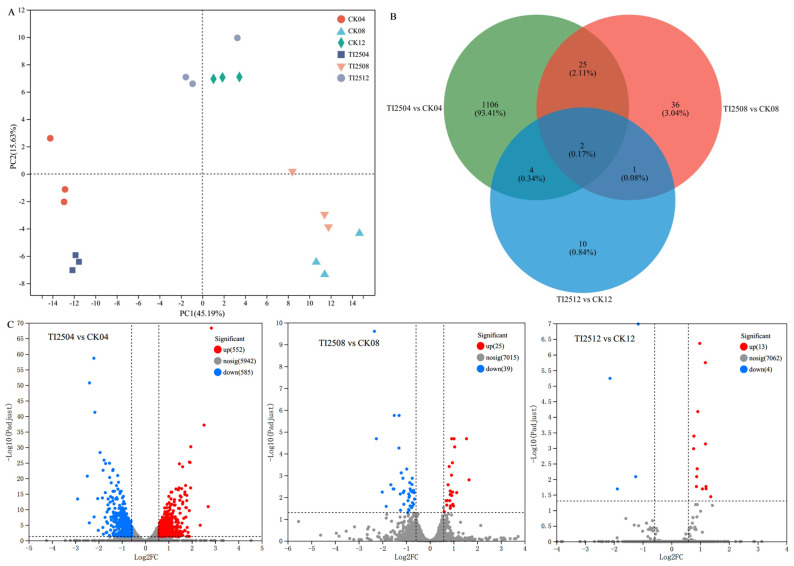
Analysis of differentially expressed genes in spore samples from different groups. (**A**) PCA graph of spore samples. (**B**) Venn diagram of differentially expressed genes in spore samples of each group. (**C**) Volcano plots of gene expression differences among spore samples from different groups. Red represents upregulated genes and blue represents downregulated genes.

Principal component analysis (PCA) was conducted on the sequencing data from six groups of *M. pilosus* spore samples. The small within-group sample distance for each group indicated minimal within-group differences and good biological repeatability. At the same fermentation time, the TI-25 and CK groups were significantly separated (Figure 6A), suggesting that *mok I* gene overexpression led to significant differences in gene expression between the transformed strain TI-25 and the original strain CICC 5045.

The Venn diagram in Figure 6B shows the distribution of differentially expressed genes between the transformed strain TI-25 and the original strain CICC 5045 across different comparison sets. The differentially expressed genes were primarily concentrated in the TI2504 vs. CK04 comparison, with 1137 genes. The number of differentially expressed genes in TI2508 vs. CK08 was 64, while TI2512 vs. CK12 had only 17 differentially expressed genes.

Figure 6C shows the volcano plot of differentially expressed genes between the transformed strain TI-25 and the original strain CICC 5045 at the same fermentation time. In the TI2504 vs. CK04 comparison, 552 genes were upregulated and 585 genes were downregulated; in the TI2508 vs. CK08 comparison, 25 genes were upregulated and 39 genes were downregulated; and in the TI2512 vs. CK12 comparison, 13 genes were upregulated and four genes were downregulated.

#### 3.6.2. EggNOG, GO, and KEGG Functional Annotation Analysis of Differentially Expressed Genes in Transformed Strains

**Figure 7:**
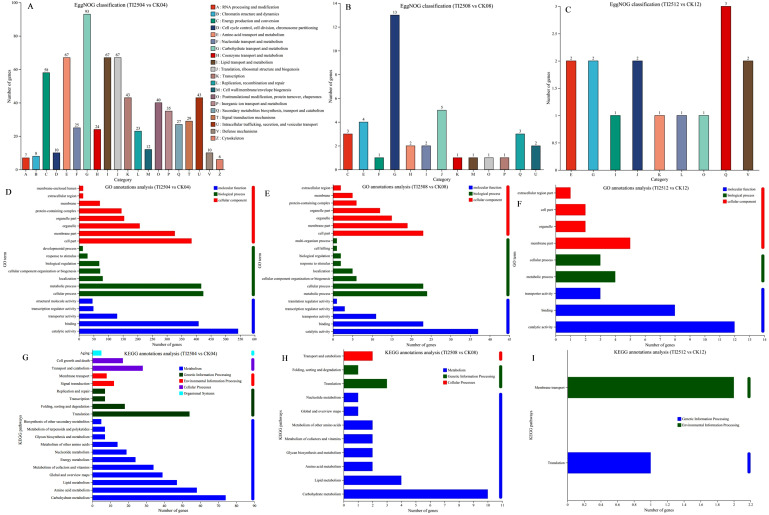
GO, EggNOG, and KEGG functional annotation analysis of differentially expressed genes in spore samples from different groups. (**A**–**C**) EggNOG functional annotation. (**D**–**F**) GO functional annotation. (**G**–**I**) KEGG functional annotation. (**A**,**D**,**G**) TI2504 vs. CK04. (**B**,**E**,**H**) TI2508 vs. CK08. (**C**,**F**,**I**) TI2512 vs. CK12.

The EggNOG functional annotation analysis results (Figure 7A–C) showed that differentially expressed genes in TI-25 vs. CK were mainly concentrated in the following categories: carbohydrate transport and metabolism, amino acid transport and metabolism, lipid transport and metabolism, translation, ribosomal structure, and biogenesis, energy production and conversion, transcription, intracellular trafficking, secretion, and vesicular transport, posttranslational modification, protein turnover, chaperones, inorganic ion transport and metabolism, signal transduction mechanisms, secondary metabolites biosynthesis, transport and catabolism, nucleotide transport and metabolism, coenzyme transport and metabolism, replication, recombination and repair, cell wall/membrane/envelope biogenesis, cell cycle control, cell division, chromosome partitioning, defense mechanisms, chromatin structure and dynamics, RNA processing and modification, and cytoskeleton, among others.

The GO functional annotation analysis results (Figure 7D–F) reveal that differentially expressed genes in TI-25 vs. CK were concentrated in the cellular component (CC), biological process (BP), and molecular function (MF) categories. In the CC category, genes were mainly associated with the cell part, membrane part, organelle, protein-containing complex, organelle part, and membrane. In the BP category, genes were primarily linked to the cellular process, the metabolic process, localization, cellular component organization or biogenesis, biological regulation, the response to stimulus, and the developmental process. In the MF category, genes were mainly associated with catalytic activity, binding, transporter activity, transcription regulator activity, and structural molecule activity.

The KEGG functional annotation analysis results (Figure 7G–I) indicate that differentially expressed genes in TI-25 vs. CK were primarily concentrated in KEGG pathways related to metabolism, genetic information processing, cellular processes, environmental information processing, and organismal systems. In the metabolism category, genes were predominantly associated with carbohydrate metabolism, amino acid metabolism, lipid metabolism, global and overview maps, the metabolism of cofactors and vitamins, energy metabolism, nucleotide metabolism, the metabolism of other amino acids, the metabolism of terpenoids and polyketides, glycan biosynthesis and metabolism, and the biosynthesis of other secondary metabolites.

#### 3.6.3. GO and KEGG Functional Enrichment Analysis of Differentially Expressed Genes in Transformed Strains

**Figure 8:**
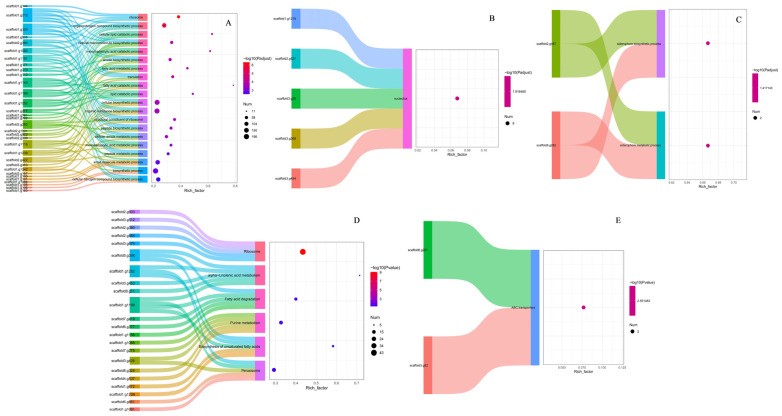
Sankey-bubble diagram for GO and KEGG enrichment analysis of differential genes in spore samples from different groups. (**A**–**C**) GO enrichment. (**D**–**E**) KEGG enrichment. (**A**,**D**) TI2504 vs. CK04. (**B**) TI2508 vs. CK08. (**C**,**E**) TI2512 vs. CK12. The top 20 enrichment pathways were showed in the bubble chart, and the top 5 differential genes were showed in the Sankey plot sorted based on the *p*-value.

The Sankey-bubble diagrams in Figure 8A–C illustrate the main pathways of GO functional enrichment for differential genes in TI-25 vs. CK (*p*-value < 0.05), as well as the primary differentially expressed genes in each pathway. The differential genes in ti-2504 vs. ck04 were predominantly enriched in pathways related to the biosynthetic process, small molecule metabolic process, peptide metabolic process, monocarboxylic acid metabolic process, cellular amide metabolic process, peptide biosynthetic process, structural constituent of ribosome, organic substance biosynthetic process, cellular biosynthetic process, lipid catabolic process, fatty acid catabolic process, translation, fatty acid metabolic process, amide biosynthetic process, monocarboxylic acid catabolic process, cellular macromolecule biosynthetic process, cellular lipid catabolic process, organonitrogen compound biosynthetic process, ribosome, and cellular nitrogen compound biosynthetic process (Figure 8A). The differential genes in TI-2508 vs. CK08 were mainly enriched in the nucleolus pathways (Figure 8B). The differential genes in TI-2512 vs. CK12 were primarily enriched in pathways related to the siderophore biosynthetic process and siderophore metabolic process (Figure 8C).

The Sankey-bubble diagrams in Figure 8D,E display the main pathways of KEGG functional enrichment for differential genes in TI-25 vs. CK (*p*-value < 0.05), as well as the key differentially expressed genes in each pathway. The differential genes in TI-2504 vs. CK04 were mainly enriched in KEGG pathways related to ribosome, alpha-linolenic acid metabolism, fatty acid degradation, purine metabolism, the biosynthesis of unsaturated fatty acids, and peroxisome (Figure 8D). The differential genes in TI-2512 vs. CK12 were primarily enriched in KEGG pathways related to ABC transporters (Figure 8E). However, the differentially expressed genes in TI-2508 vs. CK08 did not show significant enrichment in any KEGG pathways (*p*-value < 0.05).

### 3.7. Metabolomic Analysis Results of the Molecular Mechanism of High MK Production in Transformed Strains

#### 3.7.1. Metabolome Detection Results and Multivariate Statistical Analysis of RYR Samples

The non-targeted metabolomics detection results based on LC-MS showed that 4098 ion peaks were detected, and 1350 metabolites were identified in positive ion mode across all RYR samples from the transformed strain TI-25 and the original strain CICC 5045 at 4, 8, and 12 days of fermentation. In negative ion mode, 4696 ion peaks were detected, and 1053 metabolites were identified.

**Figure 9:**
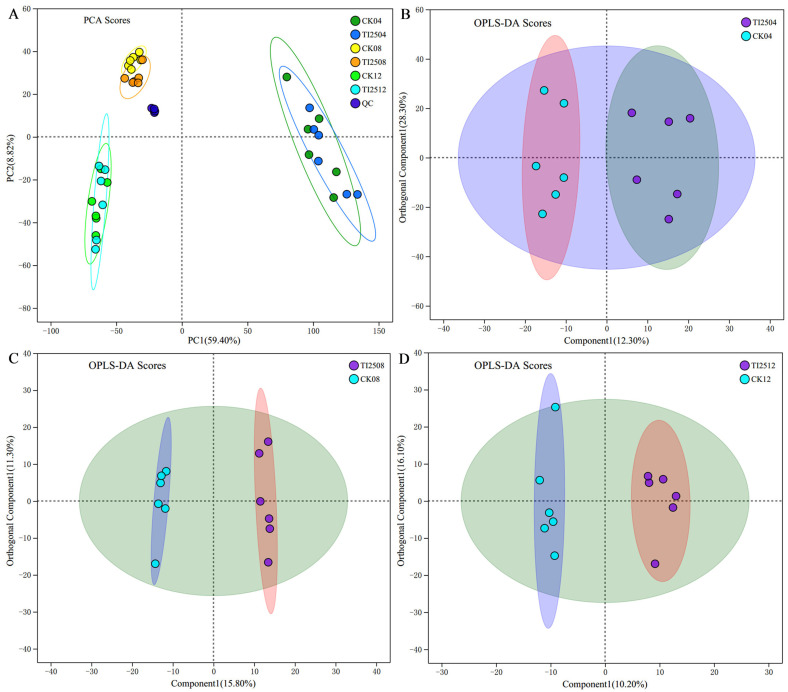
PCA and OPLS-DA analysis of metabolites in RYR samples. (**A**) PCA score chart. (**B**–**D**) OPLS-DA score charts of TI2504 vs. CK04, TI2508 vs. CK08, and TI2512 vs. CK12.

The PCA and OPLS-DA (orthogonal partial least squares discriminant analysis) scoring results for metabolites in RYR samples are shown in Figure 9A–D. The separation between the TI2508 group and the CK08 group was more pronounced compared to the other two comparison groups, indicating that there were more differential metabolites between the transformed strain TI-25 and the original strain CICC 5045 at 8 days of fermentation. In contrast, there were relatively fewer differential metabolites at 4 and 12 days of fermentation.

#### 3.7.2. Differential Metabolites and Pathway Enrichment Analysis of RYR Samples

**Figure 10:**
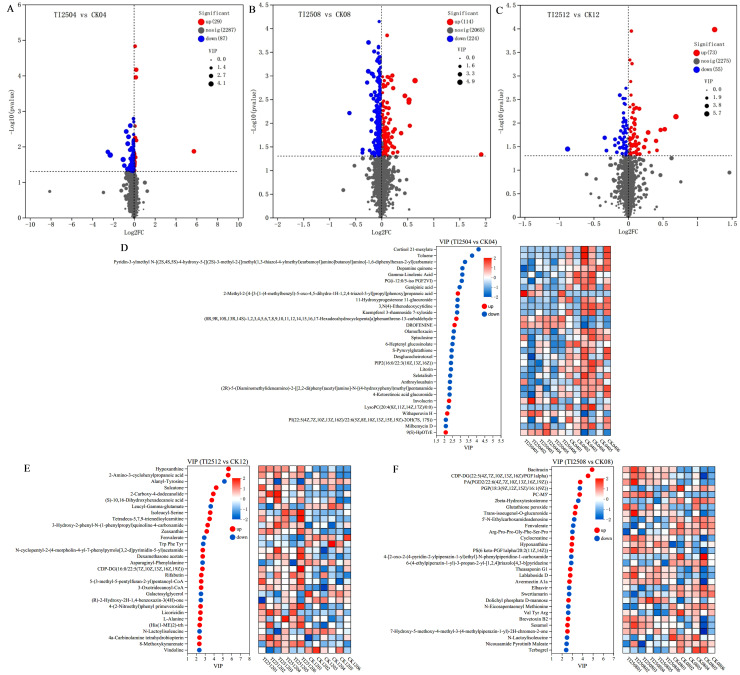
Volcano plot and VIP value analysis of differential metabolites in RYR samples from different groups. (**A**–**C**) Volcano plots. (**D**–**F**) VIP value analysis charts. (**A**,**D**) TI2504 vs. CK04. (**B**,**E**) TI2508 vs. CK08. (**C**,**F**) TI2512 vs. CK12. The top 30 metabolites were showed in the VIP chart sorted based on the VIP value. Red represents upregulated metabolites and blue represents downregulated metabolites.

Figure 10A–C displays the volcano plots of differential metabolites between the transformed strain TI-25 and the original strain CICC 5045 at the same fermentation times. There were 116 differential metabolites in TI2504 vs. CK04, including 29 upregulated metabolites and 87 downregulated metabolites. In TI2508 vs. CK08, there were 338 differential metabolites, comprising 114 upregulated metabolites and 224 downregulated metabolites. In TI2512 vs. CK12, there were 128 differential metabolites, with 73 upregulated and 55 downregulated.

Figure 10D–F shows the VIP (variable importance in the projection) value analysis of differential metabolites between the transformed strain TI-25 and the original strain CICC 5045 at the same fermentation times. In TI2504 vs. CK04, six metabolites were upregulated and 24 were downregulated. In TI2508 vs. CK08, 16 metabolites were upregulated and 14 were downregulated. In TI2512 vs. CK12, 21 metabolites were upregulated and nine were downregulated.

**Figure 11:**
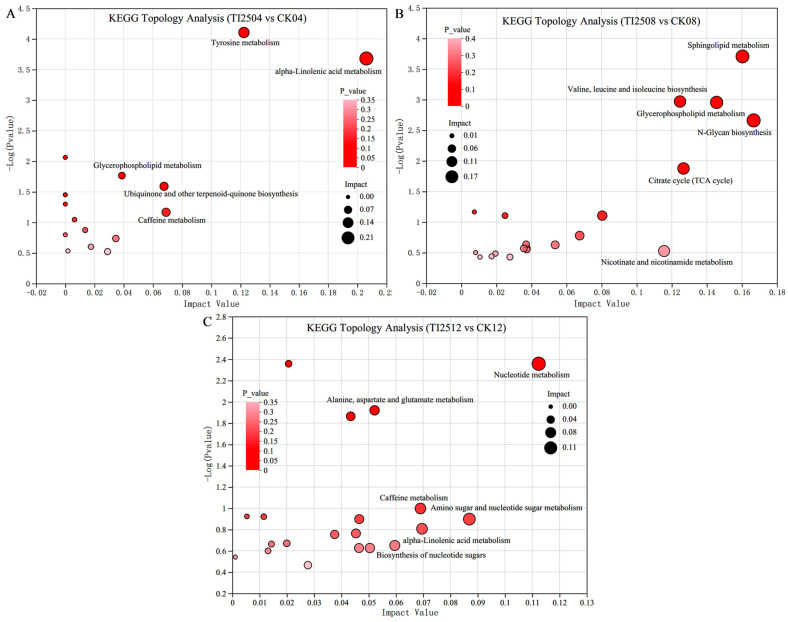
KEGG topology analysis of differential metabolites in RYR samples from different groups. (**A**) TI2504 vs. CK04. (**B**) TI2508 vs. CK08. (**C**) TI2512 vs. CK12. The top 5–6 enrichment pathways were labeled in the bubble charts based on the impact value and *p*-value.

Metabolic sets were created from the differential metabolites in the RYR samples of the transformed strain TI-25 and the original strain CICC 5045, and a topological analysis of KEGG pathway enrichment on the metabolic sets was performed. The results show that the differential metabolites in TI2504 vs. CK04 are mainly enriched in pathways linked to alpha-linolenic acid metabolism, tyrosine metabolism, caffeine metabolism, ubiquinone and other terpenoid-quinone biosynthesis, and glycerophospholipid metabolism (Figure 11A). In TI2508 vs. CK08, the differential metabolites are primarily enriched in pathways linked to N-glycan biosynthesis, sphingolipid metabolism, glycerophospholipid metabolism, the citrate cycle (TCA cycle), valine, leucine, and isoleucine biosynthesis, and nicotinate and nicotinamide metabolism (Figure 11B). In TI2512 vs. CK12, the differential metabolites are mainly enriched in pathways linked to nucleotide metabolism, amino sugar and nucleotide sugar metabolism, alpha-linolenic acid metabolism, caffeine metabolism, the biosynthesis of nucleotide sugars, and alanine, aspartate, and glutamate metabolism (Figure 11C).

### 3.8. Multi-Omics Joint Analysis Results of the Molecular Mechanism of High MK Production in Transformed Strains

**Figure 12:**
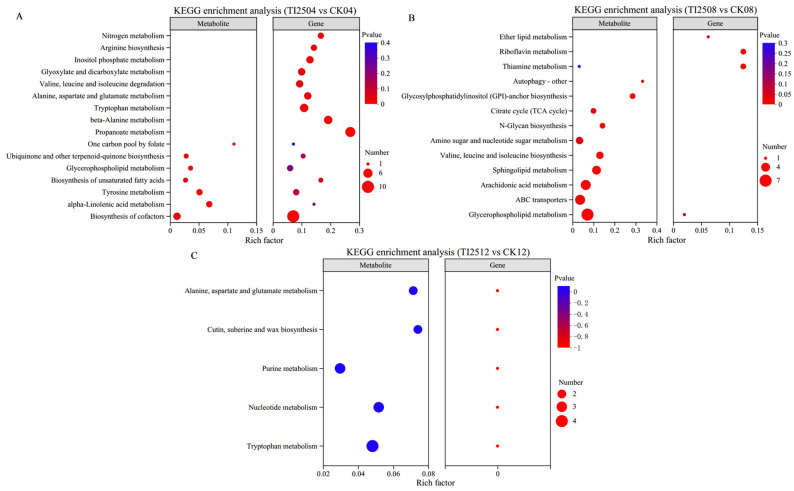
Enrichment map of KEGG pathway between differentially expressed genes in spore samples and differential metabolites in RYR samples from different groups. (**A**) TI2504 vs. CK04. (**B**) TI2508 vs. CK08. (**C**) TI2512 vs. CK12.

The KEGG enrichment results (Figure 12) from the joint analysis of transcriptomics and metabolomics indicate that the differentially expressed genes and metabolites in TI2504 vs. CK04 and TI2508 vs. CK08 are mainly co-enriched in the following pathways: the biosynthesis of cofactors, the biosynthesis of unsaturated fatty acids, tyrosine metabolism, ubiquinone and other terpenoid-quinone biosynthesis, alpha-linolenic acid metabolism, and glycerophospholipid metabolism. In contrast, the differential genes and metabolites in TI2512 vs. CK12 do not show co-enrichment in any pathway.

Among the six metabolic pathways mentioned, the following metabolites were upregulated: delta-tocopherol, alpha-linolenic acid, and 9(S)-HpOTrE. The downregulated metabolites included homogentisic acid, glutathione disulfide (GSSG), coproporphyrinogen III, gamma-linolenic acid, 4-hydroxy-phenylethanol, 3,4-dihydroxy-phenylacetate, gentisate, 9-oxononanoic acid, 1-acyl-sn-glycero-3-phosphocholine, and phosphatidyl-L-serine. Additionally, the following genes were upregulated: *monoamine oxidase* (*MAO*), *aspartate aminotransferase* (*AAT*), *4-hydroxyphen-ylpyruvate dioxygenase* (*HPD)*, *phospholipase C (PLC)*, and *CDP-diacylglycerol-inositol 3-phosphatidyltransferase* (*CDIPT*). Conversely, the genes *4-coumarate-CoA ligase* (*4CL*), *acyl-CoA oxidase* (*ACX*), and *phosphatidylserine decarboxylase* (*PSD*) were downregulated.

## 4. Discussion

The qRT-PCR results of this study indicated that at the same fermentation time, the expression levels of *mok I* in the transformed strains TI-13, TI-24, and TI-25, which exhibited high MK production, were significantly higher than those in the original strain CICC 5045. The increase ranged from 0.5 to 10.9 times. This suggests that the overexpression of *mok I* was successful, leading to enhanced MK synthesis in *M. pilosus* and a significant increase in MK production in the overexpressed strains. Other studies have also explored various strategies to improve MK production in *Monascus*. For instance, Yang et al. [37] used non-ionic surfactants as extractants in an extraction fermentation system, leading to increased expression of *mok I* and improved MK production. Similarly, the addition of γ-butyrolactone in *M. purpureus* resulted in increased transcription levels of various genes, including, including *mok I* [38]. Chen et al. [11,17] concluded that the expression levels of *mok D, mok E, mok G, mok H*, and *mok I* were positively correlated with MK production.

There have been previous research reports on improving the MK production of *Monascus* species through genetic engineering. Zhang et al. [19] increased the MK production of *M.* purpureus by 10% compared to the original strain by overexpression of the *mok I* gene. In this study, the overexpression of the *mok I* gene in *M. pilosus* resulted in a significant increase (41.39%) in MK production, which was superior to the results of Zhang et al. Hong et al. [25] increased the MK yield of *M. pilosus* by 45.73% compared to the control strain by overexpression of the *mok I* gene, based on the knockout of the citrinin synthesis regulatory gene *ctn R*. Li et al. [39] significantly enhanced the expression of almost all genes responsible for MK biosynthesis in *M. pilosus* by blocking the competitive pathway of MK biosynthesis, increasing the supply of precursors for MK biosynthesis, and implementing strategies such as histone acetylation modification, resulting in a 43.9% increase in MK production. The increases in MK production in these two studies are comparable to that of our study, but the research strategies they adopted are more complex and require multi-step metabolic engineering to achieve the research objectives.

Although some scholars have conducted research on the overexpression of the *mok I* gene to increase the MK production of *Monascus* species [19,25], the molecular mechanism has not been elucidated. This study comprehensively analyzed the molecular mechanism by which the overexpression of the *mok I* gene in *M. pilosus* increases its MK production using transcriptomics and metabolomics techniques. The results of this study can provide a theoretical basis for further research on the metabolic regulation of MK biosynthesis in *Monascus* species, and effectively improve their MK production in the future.

The multi-omics joint analysis results of this study indicated that alpha-linolenic acid was significantly upregulated in the biosynthesis of unsaturated fatty acids pathway. Alpha-linolenic acid is a type of polyunsaturated fatty acid that is metabolized to form long-chain polyunsaturated fatty acids, namely, docosahexaenoic acid (DHA) and arachidonic acid (ARA) [40]. Kraboun et al. [41] found through metabolomics analysis that *M. purpureus* produced long-chain unsaturated fatty acids such as DHA and ARA during the fermentation process of RYR. These long-chain unsaturated fatty acids play a crucial positive regulatory role in the MK biosynthesis process of *M. purpureus* [42].

In the tyrosine metabolism pathway, genes such as *MAO*, *AAT*, and *HPD* were significantly upregulated, suggesting that the overexpression of *mok I* enhances this pathway. Qiao et al. [43] observed that the tyrosine metabolism pathway was significantly enhanced during the immobilized fermentation process of *M. purpureus*, speculating that more acetyl-CoA was produced to participate in the biosynthesis of polyketide compounds. Therefore, in this study, the enhancement of the tyrosine metabolism pathway by the overexpression of *mok I* may have led to the increased production of acetyl-CoA, which is involved in the biosynthesis of MK.

## 5. Conclusions

This study constructed seven transformed strains of *M. pilosus* overexpressing the *mok I* gene and producing high MK, which showed a relative increase in MK production of 9.63% to 41.39% compared to the original strain, and none of them detected citrinin. The expression levels of *mok I* in the transformed strains TI-13, TI-24, and TI-25 increased by more than 50% compared to the original strain at different fermentation times, with the highest increase being 10.9 times. The results of multi-omics analysis revealed that the differential genes and metabolites between transformed strains and the original strain at the same fermentation times were enriched in metabolic pathways related to the biosynthesis of cofactors, the biosynthesis of unsaturated fatty acids, tyrosine metabolism, ubiquinone and other terpenoid-quinone biosynthesis, alpha-linolenic acid metabolism, and glycerophospholipid metabolism.

In summary, the overexpression of the *mok I* gene may enhance the MK synthesis level of *M. pilosus* by regulating key metabolites (such as delta-Tocopherol and alpha-Linolenic acid) and key genes (such as *MAO*, *HPD*, *ACX*, and *PLC*) in the above six metabolic pathways.

The results of this study can lay the foundation for further research on the metabolic regulation of MK in *Monascus* species. The key genes and metabolites in the above pathways may become new regulatory targets and factors for studying the mechanism of MK biosynthesis in *Monascus* species in the future. With in-depth research on these regulatory targets and factors, it may provide a new efficient way for *Monascus* and for related industries to produce *Monascus*-fermented products rich in MK.

## Data Availability

All data generated or analyzed during this study are included in this article and its Supplementary Materials.

## Supplementary Materials

The following supporting information can be downloaded at: https://www.mdpi.com/article/10.3390/jof10100721/s1, Figure S1: Plasmid profile of the recombinant expression vector pNeo-mkI; Figure S2: PCR and digestion identification results of transformed Escherichia coli. (M1) DNA Marker DL 15000; (1) Hind III/Sac I double digestion product, (2) BamH I/Sac I double digestion product; (M2) DNA Marker DL 2000; (3) PCR product; Figure S3: PCR identification of M. pilosus transformants. (M) DNA Marker DL 2000; (1-6) Transformants of M. pilosus; (7) Positive control (plasmid vector pNeo-mkI); (8) Negative control (the original strain CICC 5045). Table S1: Primers for qRT-PCR detection of expression levels of mok I gene; Table S2: The detection results of RNA samples.
