# Supplementary material for: Molecular Mechanism of Mok I Gene Overexpression in Enhancing Monacolin K Production in Monascus pilosus
Source: J Fungi (Basel). 2024 Oct 16;10(10):721. doi: 10.3390/jof10100721 (PMC11508744; doi:10.3390/jof10100721)
Supplement: Supplementary file 1 [file jof-10-00721-s001.zip › jof-3220287-supplementary.pdf]

Journal of Fungi

MDPI

Supplementary Materials

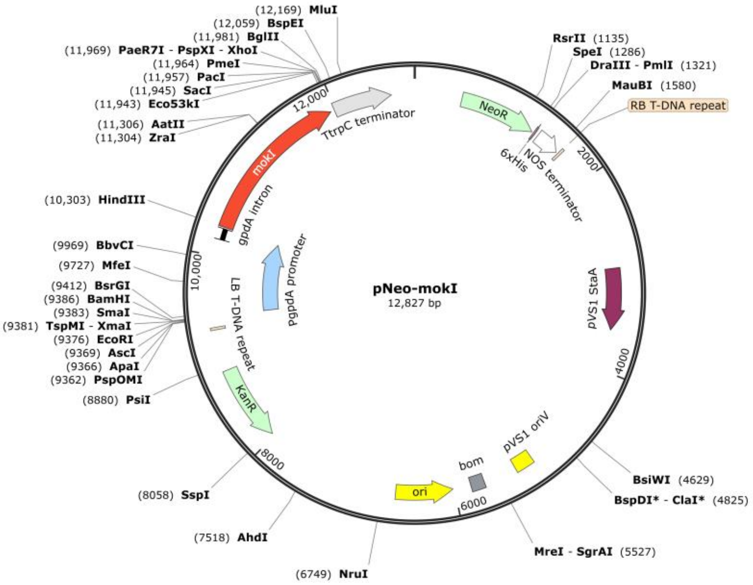

Figure S1. Plasmid profile of the recombinant expression vector pNeo-mkI.

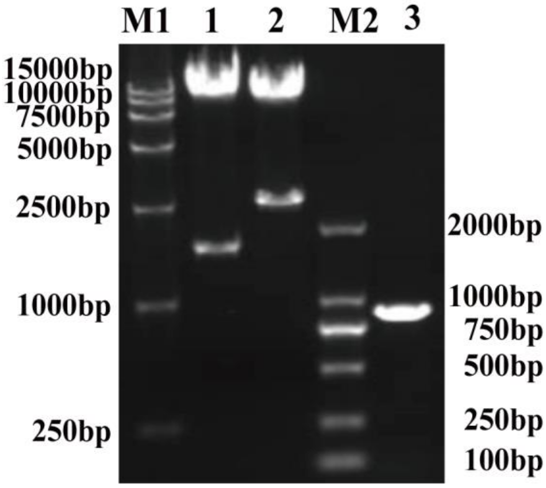

Figure S2. PCR and digestion identification results of transformed *Escherichia coli*. (M1) DNA Marker DL 15000; (1) *Hind* III/*Sac* I double digestion product, (2) *Bam*H I/*Sac* I double digestion product ; (M2) DNA Marker DL 2000; (3) PCR product.

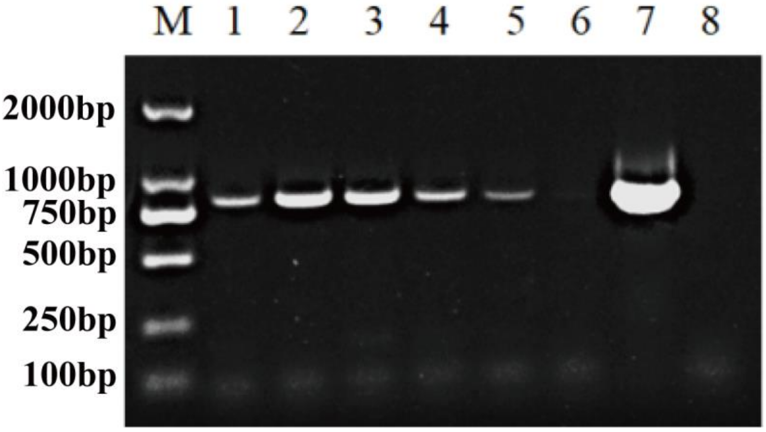

Figure S3. PCR identification of *M. pilosus* transformants. (M) DNA Marker DL 2000; (1-6) Transformants of *M. pilosus*; (7) Positive control (plasmid vector pNeo-mkI); (8) Negative control (the original strain CICC 5045).

Table S1. Primers for qRT-PCR detection of expression levels of *mok I* gene.

Genes	Primers	Sequence (5'-3')
mok I	MKI-F	CAGAACCCAAACACCACC
	MKI-R	TGATCTCACTTGCGTTAGAAT
β-Actin	Actin-F	AGTCCAACAGGGAGAAGATG
	Actin-R	CACCAGAGTCAAGCACGATA

Table S2. The detection results of RNA samples.

RNA samples	Concentration (ng/μL)	Total amount (μg)	RQN	Quality testing results
CK0401	78.47	2.75	9.60	C
CK0402	83.73	2.93	8.70	C
CK0403	74.25	2.60	9.50	C
TI250401	62.63	2.19	10.00	C
TI250402	67.50	2.36	8.60	B
TI250403	57.97	2.03	7.40	C
CK0801	27.49	0.96	10.00	B
CK0802	69.90	2.45	10.00	B
CK0803	49.04	1.72	6.80	C
TI250801	71.26	2.49	10.00	B
TI250802	28.01	0.98	9.10	C
TI250803	44.57	1.56	8.50	C
CK1201	48.14	1.69	10.00	C
CK1202	54.38	1.90	10.00	B
CK1203	47.00	1.64	10.00	B
TI251201	43.07	1.51	8.70	B
TI251202	23.52	0.82	7.00	B
TI251203	59.04	2.07	10.00	A

RQN: RNA Quality Number.
